# A computational study of cancer hyperthermia based on vascular magnetic nanoconstructs

**DOI:** 10.1098/rsos.160287

**Published:** 2016-09-14

**Authors:** Mahdi Nabil, Paolo Zunino

**Affiliations:** 1 Department of Mechanical and Nuclear Engineering, The Pennsylvania State University, University Park, PA, USA; 2 Modeling and Scientific Computing (MOX), Department of Mathematics, Politecnico di Milano, Milano, Italy

**Keywords:** cancer hyperthermia, heat and mass transfer, finite-element simulations, embedded multiscale method, nanotechnology

## Abstract

The application of hyperthermia to cancer treatment is studied using a novel model arising from the fundamental principles of flow, mass and heat transport in biological tissues. The model is defined at the scale of the tumour microenvironment and an advanced computational scheme called the *embedded multiscale method* is adopted to solve the governing equations. More precisely, this approach involves modelling capillaries as one-dimensional channels carrying flow, and special mathematical operators are used to model their interaction with the surrounding tissue. The proposed computational scheme is used to analyse hyperthermic treatment of cancer based on systemically injected vascular magnetic nanoconstructs carrying super-paramagnetic iron oxide nanoparticles. An alternating magnetic field is used to excite the nanoconstructs and generate localized heat within the tissue. The proposed model is particularly adequate for this application, since it has a unique capability of incorporating microvasculature configurations based on physiological data combined with coupled capillary flow, interstitial filtration and heat transfer. A virtual tumour model is initialized and the spatio-temporal distribution of nanoconstructs in the vascular network is analysed. In particular, for a reference iron oxide concentration, temperature maps of several different hypothesized treatments are generated in the virtual tumour model. The observations of the current study might in future guide the design of more efficient treatments for cancer hyperthermia.

## 1. Introduction

Hyperthermia is an emerging therapeutic strategy for cancer treatment. In this approach, the malignant tissue is subjected to a high temperature, falling in the range between 42°C and 50°C for a sufficiently long time. There is evidence that high temperatures can damage and kill cancer cells. Tumour shrinkage has also been observed as a consequence of this treatment [1–5]. Unlike conventional chemotherapy approaches, it has been shown that localized hyperthermia causes minimum damage to healthy body tissues [6]. Hyperthermia has mostly been used in combination with other methods of tumour treatment, e.g. chemotherapy and radiation [7–9], showing a considerable decrease in the tumour size [10,11].

Over the past decades, mathematical and computational modelling have significantly contributed to the field of hyperthermia cancer treatment (HCT). A review of the modelling approaches to HCT highlights an evolution from full-body or organ-scale lumped parameter models towards spatio-temporal modelling approaches, based on partial differential equations solved by means of advanced numerical schemes. In early studies, Jain and co-workers [12,13] presented basic lumped and distributed mathematical frameworks to estimate the distribution of the temperature during hyperthermia in healthy and neoplastic tissues of mammals. In [14], a domain-integral-equation technique was used to solve for temperature distribution in a human pelvic tumour model under an electromagnetic field stimulation. The numerical results highlighted the feasibility of an effective localized heating of the tumour tissue. A power deposition model along with the bio-heat transfer equation was proposed in [15] to elucidate the comparative, prospective, concurrent and retrospective modalities of thermal dosimetry. The model was capable of handling scenarios with different environments and tissue thermal properties to predict the spatio-temporal temperature distribution. The results revealed the significance of mathematical modelling for clinical tumour hyperthermia treatment from planning to post-therapy evaluation. A three-dimensional coupled finite-element model was developed in [16] to study the temperature and power density distribution for interstitial tumour treatment. The authors discussed the sensitivity of the temperature field induced by electromagnetic heating of the inserted thermal seeds with respect to the configuration of the three-dimensional vessels in the tissue. However, modern techniques of hyperthermia have improved the efficacy of treatment by using nanotechnology. This treatment strategy has recently become popular since it provides localized and minimally invasive tumour therapy [17].

Iron oxide nanoparticles (IONPs), gold-based nanoparticles (AuNPs) and carbon-based nanoparticles (CNPs) have been successfully tested for the localized generation of heat upon exposure to external energy fields. AuNPs and CNPs have been used in photothermal therapy of tumour tissues [1,18–20], because of their potential to absorb near-infrared light. This favourable property of AuNPs has also been studied in novel computational investigations of targeted tumour heating [21]. On the other hand, if IONPs are exposed to an alternating magnetic field (AMF), heat is produced [22]. One advantage of using IONPs is that magnetic resonance imaging (MRI) scanners can readily identify particles within the body organs [23–25]. The most important physical parameter in hyperthermic treatment is the specific absorption rate (SAR), which evaluates the amount of heat generated by applying exogenous energy sources onto the nanoparticles. There has been considerable *in vitro* research on SAR characterization [25–29]. For instance, a three-dimensional cell death model coupled with a heat transfer module was employed in [30] to assess the time evolution and spatial distribution of injured malignant cells and temperature field in human prostate tissue. The results of numerical simulations were then successfully verified against the conducted experiments on various solutions containing gold nanorods which were heated by near-infrared irradiation method.

The aim of this study is to compare the heating efficiency of different strategies for delivering magnetic particles to a tumour. More specifically, two different types of nanoparticles are considered in this study: small, 20 nm individual IONPs, which accumulate within the tumour tissue via the enhanced permeability and retention (EPR) effect; and large, sub-micrometre vascular magnetic nanoconstructs (VMNs) which accumulate within the tumour tissue by adhering firmly to the diseased vasculature. Two models are be adopted to describe IONPs and VMNs, respectively, because these vectors interact differently with the tissue. For the former, we use the model previously described by Nabil *et al*. [31]. For the latter, we develop here a new model. The proposed model features the essential characteristics to model nanoconstructs that actively interact with the vasculature, such as VMNs. Capillary leakage is addressed via a two-way coupling between the capillary vessels and the surrounding microenvironment, denoted as the *interstitial volume*. The solution of coupled flow and mass transport equations on the vascular network and in the interstitial volume enables us to capture the spatio-temporal distribution of nanoparticles. This information is essential for modelling heat generation, which directly depends on particle distribution, as shown experimentally in [32]. It should be noted that, as compared with the well-known and extensively used Pennes’ bioheat equation [33], the proposed computational framework represents a significant improvement in that it does not use a lumped spatial averaged formulation to model the contribution of blood perfusion on the heat transfer.

To describe these phenomena, we adopt the general approach to model flow, mass and heat transfer in a biological tissue using the theory of porous media combined with a multiscale approach that naturally fits to modelling microcirculation. The following references give an overview on these topics: [34–37]. One of the most significant features of the model is the ability to incorporate microvasculature configurations based on physiological data. This is made possible by an advanced computational scheme called the *embedded multiscale method* developed in [38,39] and already used in [40,41] for studying perfusion and drug delivery. More precisely, this approach involves modelling capillaries as one-dimensional channels carrying flow. Special mathematical operators are used to model the interaction of capillaries with the surrounding tissue. This approach falls within the general framework of computational multiscale methods, which appear to be successful tools for a qualitative prediction of the complex phenomena involved in nanomedicine (see [42–45] among many other examples).

Our approach is complementary to other multiscale methods that lay down their roots on mixture theory and mathematical homogenization (see [46–48] for some paradigmatic examples). Under the assumption of scale separation and locally periodic microstructure, these methods aim at determining the average properties of the macroscale (characterized by the whole tumour), in the asymptotic limit where the ratio between micro and macro characteristic scales vanishes. Rather than seeing these methods as competitors to the proposed approach, we think that they will complement each other. Indeed, the embedded multiscale method may facilitate and speed up the solution of the unit cell problems defined in the asymptotic homogenization approaches (see, for instance [48–50]). The numerical solutions obtained in this way may be processed by means of suitable averaging operators in order to evaluate the impact of microscale flow and transport parameters, as well as microvascular configuration such as tortuosity, on the perfusion properties of the whole tumour.

## 2. Models and methods

**Figure 1.**
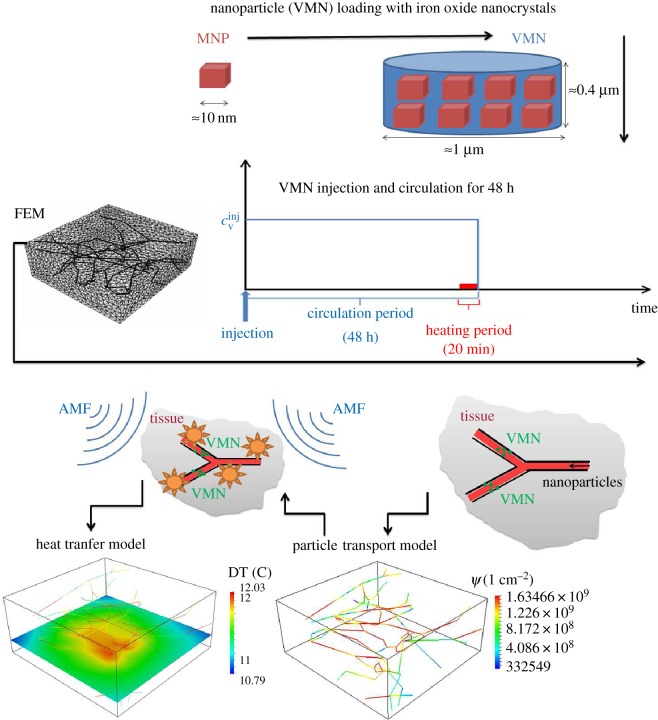
Sketch of the VMN-based hyperthermia process, split into different phases: (i) manufacturing of the particles, (ii) definition of the delivery protocol, (iii) set-up of the FEM computational model (iv) simulation of the VMN spatio-temporal distribution and (v) simulation of hyperthermia and study of the temperature maps.

We aim at studying flow in the capillary network coupled with interstitial filtration, see equation (2.1), transport of particles described by equation (2.2), generation and transfer of heat modelled by equation (2.5). As schematically described in figure 1, these are sequentially coupled phenomena, in the sense that each of them is affected by the previous ones. As a result, they have to be solved in the order they are presented here. These phenomena can be modelled by means of space–time dependent partial differential equations, which can be efficiently solved using an advanced numerical technique called the *embedded multiscale method*. This technique has been developed in [40,41] and has been adapted here to a more general setting, encompassing heat transfer. In the interest of clarity, we have discussed each model by using the following sections: assumptions, notation and governing equations, boundary and initial conditions and model parameters.

The adopted computational geometry in the current study is exhibited in figure 1. This model illustrates a tumour slab of R3230AC mammary carcinoma on a rat model, acquired in [51] and made available to the public as a result of the Microcirculation Physiome Project [52]. The tumour slab dimensions are 220×208×92×10^−5^ m and it contains 105 different segments with radii spanning from 5.5×10^−6^ m to 33.2×10^−6^ m. However, taking into account of the radius variability in the mathematical model introduces several technical difficulties, for example, in the enforcement of mass conservation at each bifurcation of the network, which have not been addressed yet in the available computational solver. For this reason, we adopt here a constant radius *R*=7.64×10^−6^ m that is the arithmetic average of the individual radii of each segment. A generalization of the flow model to variable capillary radius is presented in [53] and will be used in forthcoming studies.

The computational model is decomposed in the microvascular network and the surrounding malignant interstitial tissue, *Λ* and *Ω*, respectively. It should be mentioned that the subscript *v* (vascular) is used to represent all the variables defined on the capillaries while the variables of the tumour tissue are labelled with *t* (tissue). Our simulation studies illustrate a protocol for HCT where the host and consequently the tumour slab is infused with a solution of particles. The injected particles enter the virtual tumour model through the inlets of the capillary network. These inlets are represented by the specific points of the capillaries intersecting with the chosen faces of the tumour slab.

### 2.1. Flow

#### 2.1.1. Assumptions

The flow model includes two components, i.e. the microcirculation and the flow in the interstitial tissue. These parts interact by means of coupling conditions at the interface. In fact, the microvascular wall is considered to behave as a semipermeable membrane. Furthermore, the malignant interstitial volume is assumed to act as an isotropic porous structure. The flow through the tumour interstitium is represented by Darcy’s filtration law.

Microcirculation is characterized by low Womersley and Reynolds numbers [54–56]. In these conditions, the general Navier–Stokes equations reduce to steady Stokes flow. Under additional assumptions of (i) straight channels, (ii) rigid walls, (iii) constant radius, (iv) no-slip boundary conditions for the velocity and (v) the absence of body forces such as gravity and inertial forces; Stokes flow further simplifies to Poiseuille equations, namely (2.1e). As discussed in [55,57–59] using the Poiseuille model considerably reduces the computational cost of the problem. However, we apply the model in conditions where Poiseuille’s assumptions are not rigorously satisfied. In particular, transmural flow is allowed, but it must be small with respect to the axial velocity component. Furthermore, each vessel branch does not have to be straight, but curvature must be small. Improvements of the current flow model are in order: (i) for a better approximation of the extravasation effects; (ii) to relieve constraints on small curvature and consequently allow to analyse configurations characterized by high tortuosity, which plays an important role on the average hydraulic conductivity of tumours, as discussed in [50]; (iii) to improve the characterization of stresses on the wall, which in turn will affect particle adhesion.

The arc length coordinate along with each capillary segment is represented by the symbol *s*, while ***λ*** denotes the reference vector that characterizes the segment orientation. Furthermore, the approach of [60,61] is applied to model lymphatic drainage.

#### 2.1.2. Notation and governing equations

The coupled mathematical model for microcirculation and flow in interstitial volume is to find the pressure *p*_*t*_, *p*_v_ and the velocity fields **u**_*t*_, **u**_v_ such that the flow problem is

2.1*a* $$\begin{array}{rl} & -\nabla \cdot \left(\frac{\kappa }{\mu }\nabla p_{t}\right)+L_{p}^{\mathrm{LF}}\frac{s}{v}(p_{t}-p_{L})-f_{b}({\bar{p}}_{t},p_{v})\delta_{\Lambda }=0,\;\mathrm{in}\;\Omega , \end{array}$$

2.1*b* $$\begin{array}{rl} & u_{t}=-\frac{\kappa }{\mu }\nabla p_{t},\;\mathrm{in}\;\Omega , \end{array}$$

2.1*c* $$\begin{array}{rl} & -\frac{\pi R^{4}}{8\mu }\frac{\partial^{2}p_{v}}{\partial s^{2}}+f_{b}({\bar{p}}_{t},p_{v})=0,\;s\in \Lambda , \end{array}$$

2.1*d* $$\begin{array}{rl} & f_{b}({\bar{p}}_{t},p_{v})=2\pi RL_{p}((p_{v}-{\bar{p}}_{t})-\sigma^{p}(\pi_{v}^{p}-\pi_{t}^{p})),\;\mathrm{in}\;\Lambda , \end{array}$$

2.1*e* $$\begin{array}{rl} & {\text{u}}_{v}=-\frac{R^{2}}{8\mu }\frac{\partial p_{v}}{\partial s}\lambda ,\;s\in \Lambda \end{array}$$

2.1*f* $$\begin{array}{rl} \;\text{and}\; & -\frac{\kappa }{\mu }\nabla p_{t}\cdot n=\beta_{b}(p_{t}-p_{0}),\;\mathrm{on}\;\partial \Omega . \end{array}$$

where ${\bar{p}}_{t}$ represents an average of the interstitial pressure acting on the capillary surface, namely

$${\bar{p}}_{t}(s)=\frac{1}{2\pi R}\int_{0}^{2\pi }p_{t}(s,\theta )R\;d\theta ,$$

*θ* being the angular coordinate on the cylindrical surface representing the capillary wall.

#### 2.1.3. Boundary conditions

We have imposed a pressure gradient along the capillary vessels. Since both the inflow and outflow of the capillary vessels are located on the outer edges of the slab, we enforced a known pressure *p*_in_ on two neighbour inlet sides. On the other hand, outlet pressure *p*_out_ is assigned to the opposite faces. The pressure drop between inlets and outlets is calibrated using the following simple approach. Given an estimate of the average blood velocity in the capillary circulation, equal to 0.1 mm s^−1^ according to the data provided by Intaglietta *et al*. [62], we use Poiseuille’s Law to calculate the corresponding pressure drop. More precisely, we have applied a fictitious model consisting of a straight rigid pipe of length |*Λ*| and radius *R*, to calculate the pressure drop *p*_in_−*p*_out_ that corresponds to a velocity of 0.1 mm s^−1^ in the pipe. We have imposed the Robin-type boundary conditions (2.1f) for the blood flow in the interstitial volume. In this equation, *p*_0_ stands for the far field pressure value, while *β*_*b*_ denotes an effective flow conductivity accounting for the tissue layers surrounding the tumour slab.

#### 2.1.4. Constitutive laws and parameters

Let *L*_*p*_ be the hydraulic permeability of the vessel wall (see table 1 for units and physiological values) and let $p_{v}-{\bar{p}}_{t}$ be the pressure difference between the vessels and the interstitial volume. Because of osmosis, the pressure drop across the capillary wall is affected by the difference in concentration of the chemicals dissolved in blood, [63,64], which determine the oncotic pressure jump $\left(\pi_{v}^{p}-\pi_{t}^{p}\right)$ modulated by the sieving coefficient *σ*^*p*^. The oncotic pressure is defined as *π*=*R*_*g*_*Tc*, where *c* is the concentration of a given osmotic agent, *R*_*g*_ is the universal gas constant and *T* stands for the absolute temperature. The coefficient *σ*^*p*^ accounts for the difference of a semipermeable membrane compared to the case of ideal permeability (i.e. no resistance force on the molecules passing through the membrane). It spans from 0 to 1, where small values characterize ideal membranes, while larger values are typical of selective filters.

**Table 1.** Parameters and data for mass and thermal transport.

symbol	parameter	units	value	source	equation
*μ*	blood viscosity	kg (ms)^−1^	4×10^−3^	[40]	(2.1*b*)
*L*_p_	capillary permeability	(m^2^s) kg^−1^	10^−10^	[40]	(2.1*d*)
LpLFs0v0	lymphatic permeability	(mmHg hour)^−1^	0.5	[40]	(2.1*a*)
*d*^IONP^	diameter of IONP	m	1×10^−8^	[32]	—
*d*^VMN^	diameter of VMN	m	0.84343×10^−6^	[65]	(2.2*c*)
*m*^IONP^	mass of IONP	gr	8×10^−18^	[32]	—
*m*^VMN^	mass of VMN	gr	5.6548×10^−13^	[65]	(2.4)
*ρ*	tissue density	kg mm^−3^	1060×10^−9^	[32]	(2.5*a*)
*γ*	tissue-specific heat capacity	J kg^−1^ K^−1^	3470	[32,34]	(2.5*a*)
*κ*	tissue thermal conductivity	kg mm^−1^ K^−1^	0.51×10^−3^	[32,34]	(2.5*a*)
*β*_T_	heat exchange coefficient	W mm^−2^ K^−1^	2×10^−5^	[32]	(2.5*b*)
*T*_bl_	blood temperature	K	273.15+37	[32]	(2.5*a*)
SAR	specific absorption rate	W kg^−1^	1×10^6^	[32]	(2.5*c*)
—	mass of Fe_3_O_4_ per VMN	gr/#	2.51328×10^−13^	[32]	(2.4)

In healthy capillaries, proteins dissolved into the blood serum and in particular albumin are responsible for most of the oncotic pressure, which can rise up to about 25 mmHg. To model this effect, we set *π*^p^_*v*_=25, *π*^p^_*t*_=0 mmHg and *σ*^p^≃1, since proteins hardly leak through the capillary walls as capillary fenestrations are small with respect to protein’s molecular radius. As a result, we obtain $\sigma^{p}(\pi_{v}^{p}-\pi_{t}^{p})=25$ mmHg (1 mmHg = 133.322 Pa). Since albumin serum concentration is 5–10 times bigger than the VMN systemic concentrations reached after injection, the oncotic effect generated by the injected VMNs can be neglected. This is the scenario taken into account in the simulations presented in this work. However, data reported in [66] show that in leaky tumour vasculature, the oncotic pressure may be significantly lower than 25 mmHg, because the ratio between the protein radius and the radius of the capillary fenestrations decreases. This is modelled by assigning a smaller value to the sieving coefficient *σ*^p^, which can fall below 10^−3^, according to Jain *et al*. [66]. Then, the role of VMNs on osmosis may become relevant. Indeed, it may happen that the radius of VMN in use will be comparable or larger than the radius of the fenestrations in the capillary wall. In this case, VMNs may become the dominant oncotic agent in the tumour vasculature. This other scenario is not considered in this work, but will be the subject of future studies.

In order to model the capillary phenotype typically observed in tumours, we increase the magnitude of their hydraulic permeability as in [60], such that the model will account for the well-known EPR effect. To balance leakage of arterial capillaries, venous and the lymphatic systems absorb the fluid in excess. For the sake of generality, we include lymphatic drainage in the model, although the lymphatic system may be disfunctional in tumours. Following [60,61], we model them as a distributed sink term in the interstitial volume. It is assumed that the volumetric flow rate due to lymphatic vessels, *Φ*^LF^, is proportional to the pressure difference between the interstitium and the lymphatics, namely $\Phi^{\mathrm{LF}}(p_{t})=L_{p}^{\mathrm{LF}}\frac{s}{v}(p_{t}-p_{L})$, where *L*^LF^_p_ is the hydraulic permeability of the lymphatic wall, *s*/*v* is the surface area of lymphatic vessels per unit volume of tissue and *p*_*L*_ is the hydrostatic pressure within the lymphatic channels. In [31], the role of capillary permeability and lymphatics on small particle (nanometre size) distribution and hyperthermia has been thoroughly analysed, with the conclusion that the former parameter plays a dominant role. Here, the same conclusion is strengthened by the fact that VMN (micrometre size) constructs do not extravasate in the time scale of interest.

### 2.2. Mass transport model

The mass transport model governs the distribution of (micrometre size) nanoconstructs (VMN) within the microvascular network. Iron oxide is delivered by loading it into VMNs (see figure 1 for a sketch of the process). The symbol *c*_v_ denotes the mass concentration of iron oxide (mass/volume) in the vasculature.

#### 2.2.1. Assumptions

VMNs are functionalized particles able to bind to (inflammatory) receptors on the capillary walls. We assume that the particle size (≃800 nm for the simulations considered here) is on average larger than the transvascular gap size, which is estimated to range between 200 and 1200 nm according to Hobbs *et al*. [67], in various types of tumours. As a result, VMNs do not extravasate, but they usually circulate for several hours in the systemic vasculature before being metabolized. Furthermore, it is assumed that injecting VMNs into the circulation does not alter the blood properties. We also posit that VMNs are not damaged during circulation and that they do not release any sub-products. Then, we assume that a fixed constant concentration of VMNs is available in the vasculature for a fixed period of time. This time window is the one that matters for HCT and it will be called from now on the circulation time. We will consider various computational experiments characterized by different circulation times.

The adhesion of VMNs to the capillary walls is described following the lines of [65,68,69]. More precisely, at each point of the vascular tree, we define a vascular adhesion rate *Π*(*s*) that directly depends on the particle size and inversely depends on the particle drag force induced by blood flow, as described in the formula for the probability of adhesion, $P_{a}$, reported below. The density of iron oxide on the vascular walls, *Ψ*(*s*,*t*), is determined by the combination of the particle concentration (in terms of iron oxide mass per volume, *c*_v_) with the vascular adhesion rate.

#### 2.2.2. Notation and governing equations

We denote by *c*_v_ the concentration of iron oxide carried by VMNs. The concentration *c*_v_ is governed by the following equations:

2.2*a* $$\begin{array}{rl} & \frac{\partial c_{v}}{\partial t}+\frac{\partial }{\partial s}\left(({\text{u}}_{v}\cdot \lambda )c_{v}-D_{v}\frac{\partial c_{v}}{\partial s}\right)+\frac{2\pi R}{\pi R^{2}}\Pi c_{v}=0,\;\mathrm{in}\;\Lambda \times (0,t), \end{array}$$

2.2*b* $$\begin{array}{rl} & P_{a}=m_{l}K_{a}^{0}\alpha_{2}\pi r_{0}^{2}\exp\left(-\beta \frac{\mu |\mathrm{WSR}|}{\alpha_{2}}\right), \end{array}$$

2.2*c* $$\begin{array}{rl} & \Pi =P_{a}|\mathrm{WSR}|\frac{d^{\mathrm{VMN}}}{2} \end{array}$$

2.2*d* $$\begin{array}{rl} \;\text{and}\; & \Psi (s,t)=\int_{0}^{t}\Pi c_{v}(s,\tau )\;d\tau . \end{array}$$

The system of equations (2.2) is complemented by the definition of the mean iron oxide concentration in the tumour slab

2.3 $$c_{\mathrm{ref}}=|\Omega {|}^{-1}\int_{\Lambda }(2\pi R\Psi +\pi R^{2}c_{v}).$$

#### 2.2.3. Boundary and initial conditions

Given a desired mean concentration, *c*_ref_, we determine the constant systemic concentration, *c*_inj_, that is necessary to match the targeted *c*_ref_ at a time equal to the particle circulation time. The concentration *c*_inj_ measures the mass of iron oxide per unit volume of blood, and it is the value that we enforce at the inlets of the virtual tumour slab, in order to make (2.2) solvable. The method for calculating *c*_inj_ on the basis of *c*_ref_ will be discussed later on. Once *c*_inj_ has been found, we also determine how many VMNs should be injected to guarantee this iron oxide concentration, that is *ρ*_inj_. This calculation is performed by means of the following conversion formula:

2.4 $$c_{\mathrm{inj}}=\rho_{\mathrm{inj}}\times \text{mass of iron oxide per VMN}.$$

#### 2.2.4. Model parameters

For VMN delivery, particle density is assumed to be equal to the density of water. The value of VMN vascular deposition parameter *Π*=3×10^−9^ m s^−1^ is calculated on the basis of constants *α*_2_=4×10^9^ #/m^2^, *β*=2.3×10^11^ N^−1^ and $m_{l}K_{a}^{0}r_{0}^{2}=1.26\times 10^{-9}$ m^2^, which come from [65], and an average wall shear rate |WSR|=50 s^−1^. This wall shear rate value is consistent with the Poiseuille flow model, namely |WSR|=4*u*_v_/*R*, using an average blood velocity in the network of 0.1 mm s^−1^ and the capillary radius of *R*=7.64 × 10^−6^ m. The extension of the model to variable wall shear rate and vascular adhesion parameter will be addressed together with one for variable capillary radius presented in [53].

### 2.3. Heat transfer model

#### 2.3.1. Assumptions

According to the general framework proposed in [36], heat transfer in a biological tissue is governed by thermal conduction and convection in the tissue matrix, blood-tissue heat exchange, exogenous heat generation and heat flux through the domain boundaries. As thoroughly studied in [32], heat is generated upon irradiation of iron oxide with exposure to a low-frequency alternating magnetic field (AMF). This phenomenon is defined through a source term, i.e. *f*_T_(*c*_v_), of the heat transfer equation in the tumour slab. Low-frequency AMF is desirable because it avoids non-specific thermal generation [31,32] due to excitation of the electrolytes dissolved in the interstitial fluid or to other mechanisms [34]. Considering that in our model heat generation takes place in a small portion of the host body, it is also reasonable to postulate blood temperature homeostasis. Therefore, blood temperature *T*_bl_ is assumed to be constant.

#### 2.3.2. Notation and governing equations

We investigate the distribution of temperature field (*T*) within the tumour tissue. Based on the aforementioned assumptions, temperature is modelled using the following set of relations. These equations include convection and diffusion of heat through interstitial flow, *T***u**_*t*_−(*κ*/*ργ*)∇*T*, as well as heat sink by blood flow, 2*πRβ*_T_(*T*−*T*_bl_), lymphatic drainage, *L*^LF^_p_(*s*/*v*)(*p*_*t*_−*p*_L_)(*T*−*T*_bl_), and also heat loss from the outer boundaries of the tumour slab, *β*_T_(*T*−*T*_bl_),

2.5*a* $$\begin{array}{rl} & \rho \gamma \left[\frac{\partial T}{\partial t}+\nabla \cdot \left(T{\text{u}}_{t}-\frac{\kappa }{\rho \gamma }\nabla T\right)+L_{p}^{\mathrm{LF}}\frac{s}{v}(p_{t}-p_{L})(T-T_{\mathrm{bl}})\right]\\ & \;+2\pi R\beta_{T}(T-T_{\mathrm{bl}})\delta_{\Lambda }=f_{T}(c_{v}),\;\mathrm{in}\;\Omega \times (0,t), \end{array}$$

2.5*b* $$\begin{array}{rl} & \left(-\kappa \nabla T+\rho \gamma T{\text{u}}_{t})\cdot n=\beta_{T}(T-T_{\mathrm{bl}}),\;\mathrm{on}\;\partial \Omega \times (0,t\right) \end{array}$$

2.5*c* $$\begin{array}{rl} \;\text{and}\; & f_{T}(c_{v})=\mathrm{SAR}(2\pi R\Psi +\pi R^{2}c_{v})\delta_{\Lambda }. \end{array}$$

#### 2.3.3. Boundary and initial conditions

We have imposed Robin-type boundary conditions in order to account for heat transfer over the external boundaries of the slab using equation (2.5b). In this expression, *β*_T_ denotes an effective thermal conductivity considering the surrounding tissue layers around the tumour tissue. Initially, the temperature of the entire tumour slab is assigned to be equal to the reference blood temperature *T*_bl_.

#### 2.3.4. Constitutive laws and parameters

For the simulations, we have used model parameters reported in table 1. The thermo-physical properties of the tissue, i.e. density, specific heat capacity and thermal conductivity, all come from [32], as well as the values of blood temperature, size and specific absorption rate (SAR) of magnetic nanoparticles.

### 2.4. Computational solver

The finite-element method (FEM) is employed to discretize the system of equations (2.1), (2.2) and (2.5). Upon dividing the tumour interstitium and the capillary vessels domains, *Ω* and *Λ*, respectively, into tiny elements (as shown in figure 1), piecewise polynomial functions are adopted to approximate the solution of the governing equations formulated in their weak (or variational) form.

We notice that computational domains *Ω* and *Λ* are dimensionally heterogeneous. The tumour tissue is three-dimensional, whereas the capillaries are one-dimensional. The numerical scheme is based on the idea of representing the capillaries as a network of one-dimensional channels acting as concentrated sources of flow and heat, immersed into the interstitial volume. As a result, it can be classified as an embedded multiscale method.

The superior feature of the employed numerical scheme is the full independence of computational grids used to approximate the equations on the vessels and in the interstitial tissue. Therefore, any arbitrary and complex capillary network geometry can be potentially used as a test case. The theoretical features of this numerical approximation method have been extensively discussed by D’Angelo [38,39]. For the implementation of these algorithms, we have used GetFEM++, an open source finite-element library of C++ [70]. We have adopted the generalized minimal residual (GMRES) method along with incomplete-LU preconditioning in order to solve the system of discretized algebraic governing equations. The computational domain consists of 49 655 grid points and 272 872 tetrahedral elements, which is sufficient to reach the independence of the numerical results from the discretization. In fact, by examining the effect of mesh size on the simulation results, the results are insensitive to the numerical grids beyond 257 109 elements. More details of the numerical scheme are presented in [40,41].

Numerical simulations have been performed on a standard single core desktop PC. The clock time for simulations is comparable to the simulated time (i.e. a simulation that ends at 12 h requires 12 h of computational time). We believe that these figures feature significant margins of improvement, as the ongoing optimization of the computational efficiency of the software actually shows.

## 3. Results and discussion

### 3.1. Protocols for simulated experiments

We recall that IONPs (≃20 nm in size) are generally characterized by a short longevity in the circulation and the mechanism of accumulation within the neoplastic tissue is mostly via passive extravasation across the tumour fenestrations, following the well-known EPR effect [68,71]. On the contrary, VMNs are designed to circulate for longer times (up to 48 h post systemic injection) and, most importantly, to adhere on the tumour vasculature rather than passively extravasating [72].

Four different circulation times are considered for the injected nanoparticles, namely 40 min (short-term circulation); 12, 24 and 48 h (long-term circulation). The 40 min case is specifically considered for the short-lived IONPs, whereas the longer circulation times are for the VMNs. In all simulations, HCT is obtained via an external AMF, which is continuously applied only for the last 20 min of nanoparticle circulation. The average concentration of magnetic material in the tumour slab, denoted as *c*_ref_, previously introduced together with system (2.2), is one of the main factors controlling the efficacy of HCT. In particular, *c*_ref_ is fixed to be 1 mg ml^−1^ (1 mg ml^−1^=1 kg m^−3^) in all simulations, in that it matches the particle concentrations used in the experiments of Cervadoro *et al*. [32]. The injected concentration, *c*_inj_, was selected to match a desired amount of magnetic material in the system, *c*_ref_, for each given value of the nanoparticle circulation. The values of *c*_inj_ required to match the target value of *c*_ref_=1 mg ml^−1^ at the final time for all the simulation cases are listed in the first column of table 2. These numbers are calculated using the numerical simulations. More precisely, based on equation (2.2d), there should be an inverse relationship between the nanoparticle circulation time and the injected concentration of nanoparticles necessary to achieve the reference concentration of *c*_ref_=1 mg ml^−1^ in the tumour. Specifically, *Ψ*(*s*,*t*)=*Π*(*s*)*c*_v_(*s*)*t*, where *Ψ* is related to the target *c*_ref_=1 mg ml^−1^. Furthermore, if *c*_v_ and *Π* are almost uniform in space, *c*_v_(*s*)=*c*_v_, *Π*(*s*)=*Π*, it is also obtained *c*_ref_=(2*πRΠc*_v_*t*+*πR*^2^*c*_v_)|*Λ*|. Using this equation, approximate estimates of the systemic concentration required to match *c*_ref_ are determined. After performing the simulation for each test case, we have adjusted the dosage, *c*_inj_, such that the desired final VMN concentration within the slab is achieved, which then resulted in the data of table 2.

**Table 2.** Required VMN systemic concentrations, *c*_inj_, for reaching *c*_ref_= 1 mg ml^−1^ using different particle circulation times.

circ. time	*c*_inj_ (kg m^−3^)	% of *c*_v_ in *c*_ref_	% of *Ψ* in *c*_ref_
40 min	342.8	44.87	55.13
12 h	28.7	3.76	96.24
24 h	14.44	1.89	98.11
48 h	7.24	0.95	99.05

### 3.2. Description of the results

**Figure 2.**
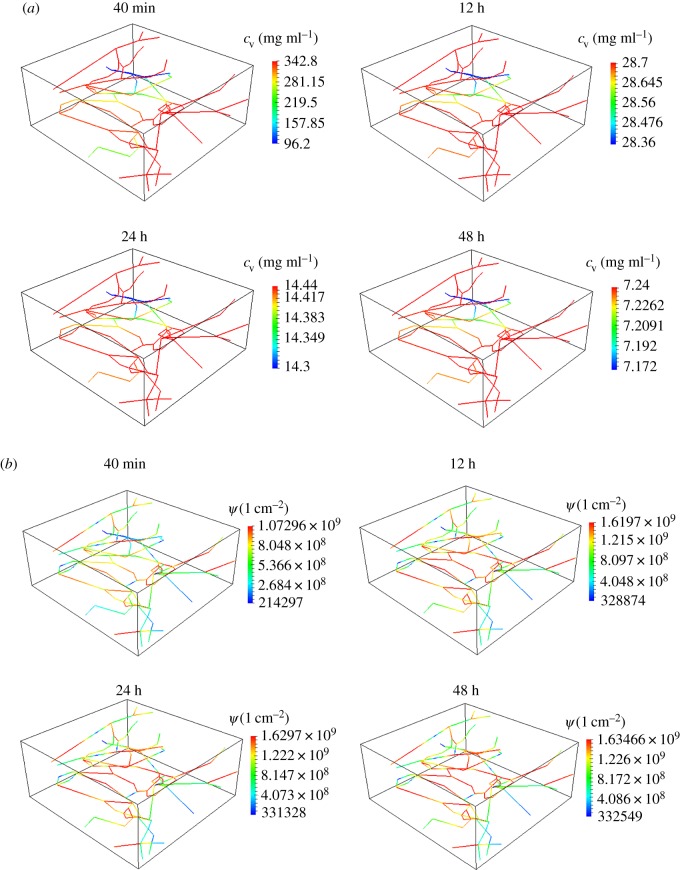
Particle concentration in the blood flow, *c*_v_, (*a*) and concentration of particles adhering to the capillary walls, *Ψ*, (*b*) for 40 min, 12 h, 24 h and 48 h of VMNs circulation time.

**Figure 3.**
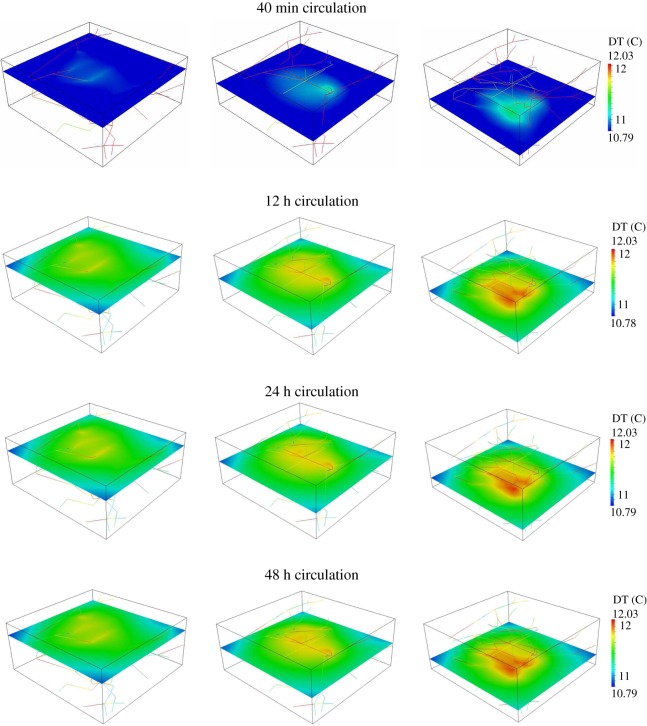
Maps of temperature increase above 37°C for 40 min, 12 h, 24 h and 48 h of VMNs particle circulation time.

**Figure 4.**
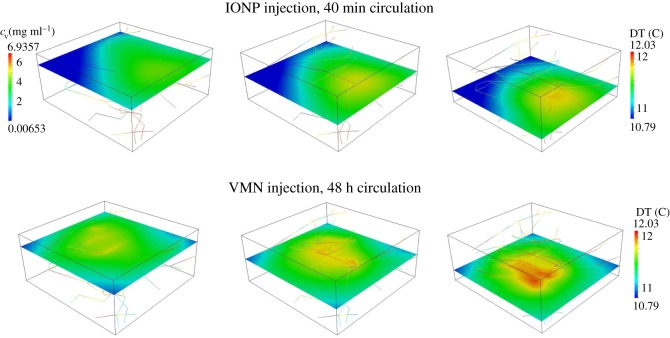
Maps of temperature increase above 37°C for IONPs injection for 40 min studied in [31] compared with VMN delivery and circulation for 48 h.

Employing the previously described computational model, the following analyses have been carried out:

(i) Quantifying the distribution of nanoparticles within the tumour as a function of the circulation times, namely 40 min, 12, 24 and 48 h. The results are presented in figure 2. In particular, figure 2*a* presents the nanoparticle concentration in the blood flow (*c*_v_), and figure 2*b* presents the density of nanoparticles adhering to the vessel walls (*Ψ*). All the cases have the same *c*_ref_=1 mg ml^−1^ at the final simulation time, which corresponds with the circulation time.
(ii) Analysis of the temperature maps associated with the AMF stimulation of VMNs as a function of the circulation times, namely 40 min, 12, 24 and 48 h. For all the tested cases, the tumour slab is exposed to an AMF only for 20 min of the circulation period. The results are shown in figure 3.
(iii) Comparison of temperature maps within the tumour slab for treatments with IONPs and VMN for a 48 h circulation time. We recall that for IONPs we do not adopt the mass transport model (2.2), but the specific model for small particles already developed in a companion study [31]. In particular, the model for IONP differs from (2.2) because it incorporates particle extravasation, diffusion and transport in the interstitial space. The temperature maps are presented in figure 4. The top panel is for the IONPs, whereas the lower panel is for VMNs.

### 3.3. Particle distribution and concentration maps

In figure 2, the volumetric concentration of nanoparticles within blood (*c*_v_) and the surface density of nanoparticles adhering to the capillaries (*Ψ*) are plotted for different circulation times. Indeed, in order to have the same *c*_ref_=1 mg ml^−1^ at the final time point, the shorter is the circulation time, the larger have to be the injected concentration of nanoparticles, *c*_inj_, and the volumetric concentration in blood, *c*_v_. This is shown in figure 2*a* where *c*_v_ steadily decreases as the circulation time increases. Note also that the spatial distribution of *c*_v_ along the vasculature is not affected by the circulation times (figure 2*a*), implying a direct scaling between vascular concentrations and circulation times. Conversely, the surface density of adhering nanoparticles grows moderately as the circulation time increases, as shown in figure 2*b*. The most significant finding is that a very large VMNs injection concentration is required to achieve the target of *c*_ref_=1 mg ml^−1^ when the short-term protocol is adopted. This delivery approach is obviously inefficient, as it does not exploit the persistence of VMNs in the circulation. By contrast, long-term delivery protocols feature much better performances, because the same target is reached with lower concentrations of injected VMNs.

Quantitative data on the volumetric concentrations and surface density of nanoparticles are provided in the third and fourth columns of table 2. The contribution of *Ψ* and *c*_v_ to the value of *c*_ref_=(2*πRΠc*_v_*t*+*πR*^2^*c*_v_)|*Λ*| at the end of circulation period is studied. As the circulation time increases, the role of *c*_v_ becomes almost negligible compared with *Ψ*. In other words, for long particle circulation time, particle-to-wall interaction (adhesion) is the main driving force for nanoparticle accumulation within the slab. Furthermore, figure 2*a* shows that for long nanoparticle circulation time, the vascular concentration of nanoparticles, *c*_v_, is almost uniform. For this reason, the approximate relation *c*_ref_≃2*πRΠc*_inj_*t* is accurate and as a result the systemic concentration, *c*_inj_, turns out to be inversely proportional to the particle circulation time. As a result, the most effective case is the 48 h, which features the longest duration.

### 3.4. Temperature maps

Temperature distribution maps are presented in figure 3. As previously mentioned, for all tested cases, the AMF exposure and in turn heat generation in the tumour tissue occurred within the last 20 min of the circulation time. First, it can be observed that temperature maps are similar in that the reference concentration of magnetic material is fixed to be *c*_ref_=1 mg ml^−1^ for all cases. In the case of 40 min, the maximum temperature reached in the slab is of 11.34°C, while for longer times it goes up to 12.03°C, only 6% higher than in the previous case.

### 3.5. Comparison of temperature maps for iron oxide nanoparticle and vascular magnetic nanoconstruct injection

In figure 4, the temperature distribution within the tumour slab for IONPs (40 min circulation) and VMNs with 48 h of circulation time are compared. In both cases, the reference concentration of *c*_ref_=1 mg ml^−1^ in the tumour slab is reached at the end of the circulation time. Similar systemic concentrations *c*_inj_ are also used in these protocols. In the VMN case, table 2 shows that *c*_inj_=7.24 kg m^−3^ of magnetic material is used in the systemic circulation, while in [31] *c*_inj_=6.94 kg m^−3^ of magnetic material were used for the simulations of IONPs in the same conditions. These data show that these two simulation protocols are almost equivalent in terms of input conditions.

For IONPs, figure 4 confirms the findings of Nabil *et al*. [31], where it is shown that for tumour slabs sufficiently large, an inhomogeneous particle distribution and temperature fields are generated. More precisely, a definite temperature gradient is observed, from the inflow to the outflow sections of the vasculature. The results reported in figure 4 for VMN show that this delivery method lowers the temperature heterogeneity in the tumour tissue. In particular, a uniform level of hyperthermia is obtained in the slab, with the highest value of temperature increase in the middle of the region of interest, where heat loss is lower compared with the tissue boundaries and the density of capillaries is higher. In conclusion, provided that the capillary network is evenly perfusing the tumour, VMN delivery guarantees uniform hyperthermia levels, rather insensitive to the direction of the microcirculation and interstitial flow.

## 4. Conclusion

A new mathematical model has been developed to investigate the coupled heat and mass transfer at the level of tumour microenvironment. Basic balance and constitutive laws have been adopted to simulate the interactions among the main compartments of the tumour tissue. The main contribution of the computational framework is that flow and heat transfer in the capillary and interstitial medium are coupled for simulations performed using a microvasculature configuration based on physiological data.

The simulations suggest that network topology and particle distribution along the microvasculature are the key factors in VMN delivery approach, because they affect the temperature field within the tumour tissue. Since VMN extravasation is unlikely, we observe that the density of particles decorating the arterial wall is directly proportional to time. This suggests that efficient delivery protocols for VMN should allow for a significant time lag between particle injection and exposure to the alternating magnetic field and consequent heating.

Finally, a comparison has been made between the 48 h VMN delivery protocol, and the case of 40 min bolus injection of iron oxide nanocrystals (IONPs). These two cases are quite similar in terms of the required injection dosage of IONPs. However, different mechanisms governing these two injection approaches result in distinct behaviours of temperature increase and different temperature distributions within the slab. In particular, for the IONP injection method, a clear temperature gradient within the slab was observed with the higher temperature occurring at the inflow section of the slab. In contrast to the IONP injection approach, the mechanisms governing VMN delivery is such that interstitial blood flow and microcirculation barely affect the level of hyperthermia. In fact, the key parameter for VMN delivery is the topology of the microvasculature. Since the capillaries spread rather uniformly within the tumour slab, a nearly constant hyperthermia level is obtained throughout the tissue.

Although the novel mathematical model provides a better understanding of the mechanisms governing tumour hyperthermia, there is still room for further improvements. For example, as previously remarked, the assumptions at the basis of Poiseuille equation are quite restrictive and they do not completely fit to microcirculation. A more detailed asymptotic analysis of Navier–Stokes equations in the limit of low Reynolds numbers may inform about better approximations of flow in highly tortuous and leaky channels. For mass transport, the contribution of VMNs to oncotic pressure gradients, which may affect the fluid balance between the vascular and interstitial compartments, should be carefully investigated. From a more general perspective, we envision two concurrent directions of development. One arises observing that in the present approach the virtual tumour model is isolated. In fact, the proposed model does not account for all the possible interactions of the malignant tissue with the surrounding microenvironment. It also does not take into account the role of tissue metabolism on the final delivered dosage of IONPs and consequently on the level of hyperthermia. Coupling the current model with other ones that account for the mass and heat metabolism at the systemic level will be a future direction of improvement. In particular, we will consider extensions of the model where the VMNs systemic concentration enforced at the endpoints of the vasculature, as well as their circulation time, are not constant but progressively decrease due to particle sequestration into surrounding organs.

The second limitation consists in the difficulty of determining the model coefficients. In particular, the interaction of VMN with the vascular wall is modelled here through the vascular adhesion parameter, which describes the macroscopic effect of many complex phenomena. A more advanced approach, currently under development, is the combination of the mass and heat transport models with accurate microscale simulations for the interplay of a single particle with blood flow and the arterial wall.

In conclusion, the present model, which addresses tumour hyperthermia through a mesoscale approach, will surely benefit from integration with macro- and micro-scale complementary models, in a comprehensive multi-scale description of VMN transport and heat generation.

## Data accessibility

This work did not involve any experimental data.
